# Cisplatin-based chemotherapy for the treatment of metastatic collecting duct carcinomas: A real-world, retrospective analysis

**DOI:** 10.3389/fonc.2022.939953

**Published:** 2022-10-18

**Authors:** Mimma Rizzo, Silvia Chiellino, Angela Gernone, Camillo Porta

**Affiliations:** ^1^ Division of Medical Oncology, Azienda Ospedaliero Universitaria (A.O.U.) Consorziale Policlinico di Bari, Bari, Italy; ^2^ Division of Medical Oncology, Istituto di Ricovero e Cura a Carattere Scientifico (I.R.C.C.S.) San Matteo University Hospital, Pavia, Italy; ^3^ Chair of Oncology, Interdisciplinary Department of Medicine, University of Bari “A. Moro”, Bari, Italy

**Keywords:** collecting duct carcinomas, non clear cell renal cell carcinoma, kidney cancer, cisplatin, chemotherapy, retrospective study

## Abstract

Collecting duct carcinomas (CDCs) are a particularly rare subtype of kidney cancer, endowed by a particularly poor prognosis. Since no active treatments have been established for CDCs, due to similarities with upper tract urothelial carcinomas, the use of the cisplatin-gemcitabine doublet is usually recommended. Here we report a retrospective analysis of 36 metastatic CDCs treated, as everyday clinical practice, with either cisplatin-gemcitabine or cisplatin-gemcitabine-paclitaxel from 2005 to 2021. Thirty-three patients received gemcitabine (1000 mg/m^2^, days 1 and 8) and cisplatin (70 mg/m^2^, day 1), while 3 were treated with paclitaxel (80 mg/m^2^, days 1 and 8), gemcitabine (1000 mg/m^2^, days 1 and 8) and cisplatin (70 mg/m^2^, day 1), every 21 days for a maximum of 6 cycles. Eight out of 36 patients (22.2%) experienced a partial response, while 9 others (25%) had a disease stabilization. No benefit was observed in the only 3 patients treated with the triplet. Median PFS was just 6 months, while median OS was 8 months. The commonest grade ≥3 treatment-related adverse events were: neutropenia (75%, 11.1% of febrile neutropenia), anemia (50%), thrombocytopenia (38.8%), and vomiting (8.3%). Dose omissions and dose reductions were common, and few frail patients started the treatment with a 25% dose reduction. In conclusion, our real-world experience confirmed the modest activity and relevant toxicity of cisplatin-based chemotherapy for the treatment of CDCs. More translational studies and novel study designs are thus badly needed in these still orphan tumors.

## Introduction

Bellini duct carcinomas, or collecting duct carcinomas (CDCs), are a particularly rare subtype of the already rare non-clear cell renal cell carcinomas, accounting for about 1% of all malignant epithelial tumors of the kidney; they arise from the renal collecting duct epithelium, and are endowed by a very poor prognosis, often presenting with a locally advanced, or frankly metastatic, disease (1).

In the United States, a relationship with African American descent, and with male sex, has been reported (2), while across different continents (America, Europe and Asia) 1- and 3-year survival rates remain constantly disheartening (2–4).

So far, no standard treatment has been established for CDCs, almost all tested agents having yielded poor results, both in terms of antitumor activity, as well as of efficacy. Due to their anatomical origin (and other biologic and morphologic features), which they do share with upper tract urothelial carcinomas, most international guidelines recommend the cisplatin-gemcitabine doublet for the treatment of metastatic CDCs (5).

In 2013 a systematic review was conducted to evaluate management options for CDCs (1); included studies had to have enrolled at least 10 subjects with histologically proven CDCs. In the same manuscript, the retrospective experience of the Mc Master University was also reported.

As a whole, a gemcitabine-cisplatin or -carboplatin regimen resulted in a 26% objective response rate (in 23 patients only), while the methotrexate-vinblastine-doxorubicin-cisplatin (MVAC) combination yielded no responses at all; similarly, old-fashion, cytokines-based, immunotherapy failed to provide any benefit (1).

Despite the conduction of a limited number of other, small, prospective studies in more recent years, CDCs remain an orphan disease, still being constantly endowed by a very poor prognosis.

Here we report a retrospective analysis of metastatic CDCs treated with cisplatin (CDDP)-based chemotherapy (either CDDP-gemcitabine or CDDP-gemcitabine-paclitaxel) at three large institutions, two from Northern, and one from Southern, Italy.

## Patients and methods

### Patients

Data from 36 previously untreated, metastatic CDC patients, treated from December 2005 to December 2021, were retrieved from original source documents (clinical charts) of the three participating centers.

As expected, there was a predominance of male patients (26/36, 72.2%), with a median age of 66.5 years (average: 65.6, range: 51-77); main metastatic sites were lymphnodes (30/36, 83.3%), lung (25/36, 69.4%), liver (22/30, 73.3%), and bones (19/30, 52.7%), more than 3 metastatic sites having been observed in all patients.

Notably, two patients only previously underwent cytoreductive nephrectomy, one having been initially misdiagnosed with a parenchymal renal cell carcinoma, and the second due to persistent macrohematuria.

Main patients’ characteristics are summarized in **Table 1**.

**Table 1:** Patients’ characteristics.

N. of patients	36	
Age (years)		
median average range	66.6 65.6 51–77	
	N.	%
Sex		
males females	26 10	72.2 27.8
Concomitant diseases		
hypertension type II diabetes chronic heart disease COPD chronic cerebral vasculopathy more than 3 comorbid conditions	30 10 10 8 5 29	83.3 27.7 27.7 22.2 13.8 80.5
Previous nephrectomy		
yes no	2 34	5.5 94.5
Baseline IMDC prognostic group		
good intermediate poor not evaluable	2 12 16 6	5.5 33.3 44.4 16.7
Number of metastatic sites at baseline		
3 >5	36 11	100 30.5
Main metastatic sites at baseline		
lymphnodes lungs liver bones	30 25 22 19	83.3 69.4 73.3 52.7
Concomitant treatments		
palliative radiotherapy bisphosphonates/denosumab embolization	13 17 1	36.1 47.2 2.7

N., number; COPD, chronic obstructive pulmonary disease; IMDC, International Metastatic RCC Database Consortium.

### Treatment

Thirty-three of the patients considered received a combination of CDDP and gemcitabine, while 3 patients only were treated with a triplet of CDDP, gemcitabine and paclitaxel, as used in the EORTC Intergroup Study 30987 for urothelial cancer patients (6).

Patients treated with the doublet were given 1000 mg/m^2^ gemcitabine on days 1 and 8, plus 70 mg/m^2^ CDDP on day 1; the two drugs were repeated every 21 days for a maximum of 6 cycles according to toxicity and efficacy.

As far as the triplet, patients were treated with paclitaxel 80 mg/m^2^, before the same doses of gemcitabine and CDDP as above; both paclitaxel and gemcitabine were administered also on day 8. Again, treatment cycles were repeated every 21 days for a maximum of 6 cycles. The use of the triplet therapy was abandoned after the implementation of local guidelines recommending the use of the cisplatin/gemcitabine doublet.

Patients had adequate hematologic, hepatic, and renal function; in particular, creatinine clearance had to be ≥60 mL/min to allow the administration of CDDP. However, since this was a retrospective analysis of real-world data, no specific inclusion/exclusion criteria were established, and co-morbid patients have been also treated.

However, all patients gave their written informed consent to treatment, according to institutional rules for everyday clinical practice.

### Activity, efficacy, and safety assessment

Response to treatment was checked after the third, and then the sixth treatment cycle (for those who completed the scheduled treatment) according to commonly used RECIST criteria. For those surviving after the end of the scheduled 6 cycles, disease status was evaluated at regular intervals (1 to 3 months). Disease evaluation was performed in all cases by means of a contrast-enhanced CT scan of thorax and abdomen; in the vast majority of patients (28/36, 77.7%), before treatment start CT scan was extended to the brain, which was not studied at 3 and 6 months unless the appearance of neurological symptoms. Bone scan was performed at baseline only in symptomatic patients, but not subsequently controlled unless considered clinically indicated.

Progression-free survival (PFS) was computed from the first day of treatment to the day of documented progression, or to the day of death from underlying cancer, whichever first, while overall survival (OS) was computed from the first day of study treatment to the day of death from any cause. PFS and OS were estimated and the relative curves plotted according to Kaplan-Meier.

As far as safety, commonest treatment-related adverse events (AEs), as well as laboratory abnormalities, were recorded and described in each patient’s source documents, from which they were retrieved, and are here summarized within **Table 2**. In light of the wide observation period considered, different versions of the National Cancer Institute Common Terminology Criteria for Adverse Events (NCI-CTCAE) were used for constitutional symptoms, whilst NCI-CTCAE version 5.0 were used to grade laboratory abnormalities, including hematological toxicities.

**Table 2:** Most common adverse events, G2 or more.

Adverse events	Grade 1 and 2		Grade 3 and 4	
*Hematological*	No. of patients	%	No. of patients	%
Anemia	19	52.7	18	50.0
Neutropenia	9	25.0	27	75.0
Febrile neutropenia			4	11.1
Thrombocytopenia	16	44.4	14	38.8
*Renal*				
Creatinine increase	10	27.7	0	0
*Gastrointestinal*				
Diarrhea	10	27.7	1	2.7
Stomatitis				
Nausea	26	72.2	0	0
Vomiting	8	22.2	3	8.3
*Other*				
Fatigue	20	55.5	1	2.7
Anorexia	18	50.0	0	0
Constipation	13	36.1	0	0
Weight loss	16	44.4	0	0
Alopecia	6	16.6		
Peripheral neuropathy	15	41.6	0	0
Hearing impairment	2	5.5	0	0
Subileus	0	0	1	2.7
Hyperglycemia	7	19.4	0	0
ALT increase	4	11.1	0	0
AST increase	3	8.3	0	0
Hyperbilirubinemia	1	2.7	1	2.7

ALT, alanine aminotransferase; AST, aspartate aminotransferase.

## Results

### Treatment activity and efficacy

**Figure 1:**
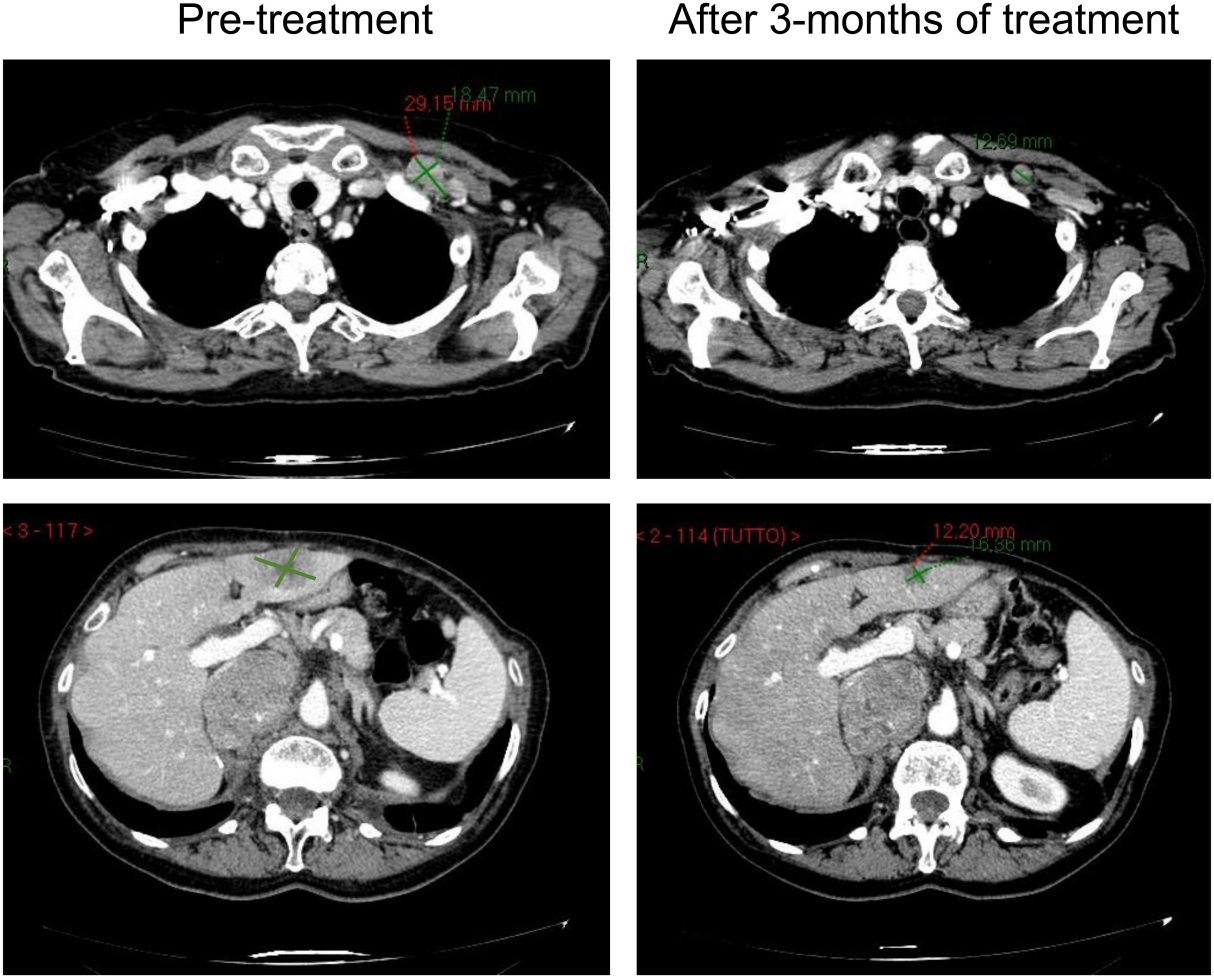
An exemplificative case of response in the liver and in a nodal lesion.

Eight out of 36 patients (22.2%) experienced a partial response, while 9 others (25%) had a disease stabilization as their best response to treatment; an exemplificative case of response (in the liver and in a nodal lesion) from cisplatin and gemcitabine chemotherapy is reported in **Figure 1**.

As a whole, disease control rate was 47.2%. Notably, none of the three patients treated with the triplet experienced any benefit (either a response or, at least, a disease stabilization).

Despite the above decent antitumor activity, median PFS was just 6 months (95% confidence interval [CI]: 4.95-6.80, average: 5.88, range: 3-15), while median OS was 8 months (95% CI: 7.40-9.89, average: 8.65, range: 3-20).

**Figure 2:**
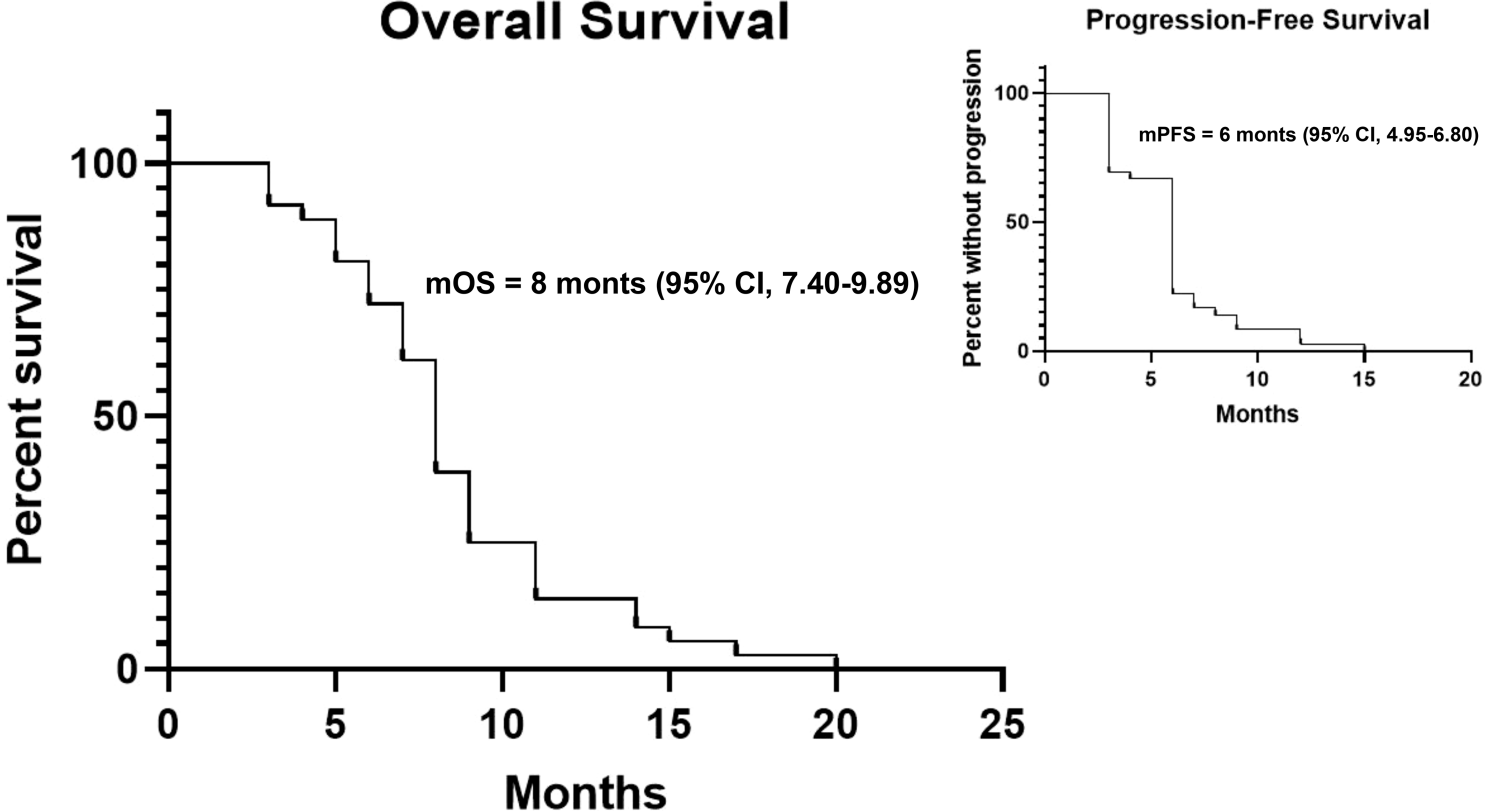
Kaplan-Meier curves for OS and PFS.

Activity and efficacy are summarized in **Table 3**, while PFS and OS curves are shown in **Figure 2**.

**Table 3:** Summary of treatment activity and efficacy.

	N.		%	95% CI
ORR	8		22.2	11.5-37.8
CR PR SD PD	0 8 9 19		0 22.2 25 52.8	- 11.5-37.8 13.7-41.0 37.2-68.2
DCR (CR + PR + SD)	17		47.2	31.7-62.7
	Median	95% CI	Average	Range
PFS	6	4.95-6.80	5.88	3-15
OS	8	7.40-9.89	8.65	3-20

N., number; ORR, overall response rate; CR, complete response; PR, partial response; SD, stable disease; PD, progressive disease; DCR, disease control rate; CI, confidence interval; PFS, progression-free survival; OS, overall survival.

### Safety

The safety profile of the two platinum-based combinations used is summarized in **Table 2**.

The most common grade ≥3 treatment-related AEs, observed in at least 5% of the treated patients, were: neutropenia (observed in 75% of the cases, with 11.1% of febrile neutropenia), anemia (50%), thrombocytopenia (38.8%), and vomiting (8.3%).

In 10 patients (27.7%), all from the CDDP/gemcitabine group, the dose of gemcitabine scheduled for the eighth day of each cycle had to be omitted, in at least one cycle, mainly due to haematological toxicity (neutropenia, febrile neutropenia, or thrombocytopenia).

As far as haematological toxicity, 8 anaemic patients (22.2%) and 14 neutropenic patients (38.8%) needed hematopoietic growth factor support with erythropoietin and granulocyte colony-stimulating factor, respectively.

All three patients treated with the triplet, as well as 2 patients treated with the doublet (5.5%) were dose reduced due to AEs; in the case of the triplet, the dose of all three agents were reduced by 25% (in one case after the first cycle, in the other two after the second), while in the case of the doublet, only the dose of CDDP was reduced (again by 25%), in both cases after the first cycle. Three patients (8.3%) from the CDDP-gemcitabine group started the treatment with a 25% dose reduction, having been considered frail; in these patients, chemotherapy dose was neither increased, nor further reduced.

## Discussion

CDCs are a particularly aggressive subset of renal malignancies, typically resistant to almost all treatments proposed so far, having nothing in common with kidney cancer’s commonest histotypes, i.e. clear cell, papillary, or chromophobe carcinomas.

The fact that CDCs are quite rare (not to take into account the difficulty in their morpho-histological diagnosis), justify why the therapeutic experiences reported in the Literature are scarce and characterized by small numbers.

CDCs are usually treated with cytotoxic chemotherapy, similarly to urothelial cancers, CDDP-gemcitabine being the most commonly used combination.

However, the results achievable by means of this combination, both in terms of activity and efficacy, are modest, at best.

In a single-arm phase II study from the French Groupe d’Etudes des Tumeurs Uro-Génitales (GETUG), the combination of gemcitabine plus either CDDP or carboplatin yielded, on 25 patients, an overall response rate (ORR) of 26%, and a median PFS and OS of 7.1, and 10.5 months, respectively (7).

More recently, in a retrospective report of 35 CDC and 22 renal medullary carcinoma patients, the three combinations of CDDP-gemcitabine, CDDP-gemcitabine and bevacizumab, or dose-dense methotrexate, vinblastine, doxorubicin and CDDP (MVAC), yielded an ORR of 26%, 41% and 56%, respectively, with a time to progression [TTP] and an OS for the whole patient population of 7.27 and 12 months, respectively (8); notably, the majority of patients (63%) received more than on line of treatment. In both cases, the platinum-based combinations proved to be feasible, being endowed by a relatively safe and easy to manage toxicity profile.

Our above experience is in line with what has been reported above. Indeed, CDDP-based combinations proved to be of limited activity and efficacy, yielding an ORR of just 22% and a median OS of 8 months only. Furthermore, this treatment proved to be fairly toxic, although feasible, in a real-world, unselected, patient population like ours, where frail and co-morbid patients were well represented, and tumor bulk was huge.

And indeed the real world nature of our report is, at the same time, the major strength and one of the weaknesses of our case series. Other limitations are the lack of a centralized review of the histological diagnosis of CDCs, the huge time span in which patients were treated (with changing attitudes towards supportive measures such as the use of haemopoietic growth factors, and unstandardized stopping rules in case of toxicity), and the possible under-reporting of treatment-related adverse events (AEs).

Cytotoxic chemotherapy has been also combined with antiangiogenics; within a single-arm phase II trial, the CDDP-gemcitabine-sorafenib combination yielded, on 26 CDC patients, an ORR of 30.8%, a disease control rate (DCR) of 84.6%, a median PFS of 8.8 months, and a median OS of 12.5 months (9). Given the good safety profile, Authors concluded that this combination “*… may be a suitable option for patients who have low Eastern Cooperative Oncology Group performance status …*” (9).

A striking median OS of 27.8 months, and a median PFS of 15.1 months were reported with the combination of gemcitabine plus CDDP (or carboplatin) and bevacizumab, but the severe toxicity observed (including two cases of pulmonary embolism), and especially the extremely low number of patients treated (only 5) represent a huge limitation of this study (10).

Beyond single case-reports or, on the other hand, large expanded access programs like the one of sunitinib (where it is difficult to extrapolate activity and efficacy of the tested agents on the few CDCs included), published data on the activity and efficacy of targeted agents are even scarcer, and – with few exceptions – not exciting in terms of results overall.

Indeed, although some Authors reported sporadic long disease stabilizations with sorafenib (11), temsirolimus (11), other (larger) studies evidenced no responses at all, and just few short-lasting disease stabilizations, mainly with sunitinib (12–14). Only recently, Procopio et al. reported the overall positive results of the prospective BONSAI phase II study in which 23 patients were treated with single-agent cabozantinib; as best overall response, 3 patients presented a SD, 1 patient achieved a CR, and 7 a PR, for an ORR of 35%; median PFS and OS were 4 and 7 months, respectively (15).

Considering that, beyond few case reports, to date immune checkpoint inhibitors have been seldom used in CDCs, it is clear that, despite the improvements achieved over the years for the treatment of metastatic RCC as a whole, these rare malignancies still remain orphan of active treatments.

Although it is reasonable to hope for some improvement with a larger use of the immune checkpoint inhibitors, given either alone or in combination, it is clear that it is necessary to invest more in translational approaches to drug development, in order to find more active treatments for these tumors. Furthermore, besides trying to rely on biomarkers, which may or may not be identified soon (realistically not so easily), CDCs – as all rare cancers – would greatly benefit from international cooperations, as well as new trial designs (e.g. adaptive or Bayesian), as already advocated (16).

As a whole, as far as the treatment of CDCs is concerned, the road ahead of us is not only still long, but also full of hurdles.

## Data availability statement

The datasets presented in this article are not readily available because of data confidentiality. Requests to access the datasets should be directed to CP.

## Author contributions

MR and CP contributed to conception and design of the study, organized the database, performed the statistical analysis, and wrote the first draft of the manuscript. All authors contributed to manuscript revision, read, and approved the submitted version.

## Conflict of interest

The authors declare that the research was conducted in the absence of any commercial or financial relationships that could be construed as a potential conflict of interest.

## Publisher’s note

All claims expressed in this article are solely those of the authors and do not necessarily represent those of their affiliated organizations, or those of the publisher, the editors and the reviewers. Any product that may be evaluated in this article, or claim that may be made by its manufacturer, is not guaranteed or endorsed by the publisher.
